# Analysis of Potential Amino Acid Biomarkers in Brain Tissue and the Effect of Galangin on Cerebral Ischemia

**DOI:** 10.3390/molecules21040438

**Published:** 2016-04-06

**Authors:** Ruocong Yang, Kun Chen, Yanyan Zhao, Pengpeng Tian, Feipeng Duan, Wenli Sun, Yuxin Liu, Zhiyong Yan, Shaojing Li

**Affiliations:** 1 Institute of Chinese Materia Medica, China Academy of Chinese Medical Sciences, Beijing 100700, China; yangruocong@163.com (R.Y.); tianpeng363@163.com (P.T.); 2 School of Life Science and Engineering, Southwest Jiaotong University, Chengdu 610031, China; 3 School of Chinese Materia Medica, Beijing University of Chinese Medicine, Beijing, 100029, China; Chenkun199202@126.com (K.C.); dfphunan111@126.com (F.D.); 4 College of Pharmaceutical Science, Hebei University, Hebei 071002, China; zhaoyany606@126.com (Y.Z.); yuxinliu@hbu.edu.cn (Y.L.); 5 Pharmacy Department, Hebei Yanda Ludaopei Hospital, Hebei 065000, China; zygcwy@163.com

**Keywords:** amino acid, galangin, biomarkers, cerebral ischemia

## Abstract

Galangin, a potent scavenger of free radicals, has been used as an herbal medicine for various ailments for centuries in Asia. With complex pathophysiology, ischemic stroke is one of the most frequent causes of death and disability worldwide. We have reported that galangin provides direct protection against ischemic injury as a potential neuroprotective agent and has potential therapeutic effects on the changes of serum amino acids in ischemic stroke; however, the mechanism of the changes of amino acids in the ischemic brain tissue has not yet been clarified. In this paper, we explored brain tissue amino acid biomarkers in the acute phase of cerebral ischemia and the effect of galangin on those potential biomarkers. Finally, we identified that glutamic acid, alanine and aspartic acid showed significant changes (*p* < 0.05 or *p* < 0.01) in galangin-treated groups compared with vehicle-treated rats and the four enzymes associated with these three AAs’ metabolic pathways; GLUD1, SLC16A10, SLC1A1 and GPT were identified by multiplex interactions with the three amino acids. By metabolite-protein network analysis and molecular docking, six of 28 proteins were identified and might become potential galangin biomarkers for acute ischemic stroke. The data in our study provides thoughts for exploring the mechanism of disease, discovering new targets for drug candidates and elucidating the related regulatory signal network.

## 1. Introduction

Galangin, a member of the flavonol class of flavonoids, is present in high concentrations in the rhizome of *Alpinia officinarum* Hance, which has been used in China for centuries as a spice and a traditional Chinese medicine for various ailments [1]. As a potent *in vitro* scavenger of free radicals such as singlet oxygen and superoxide anion [2], galangin has multiple bioactivities and affects many cell systems. In addition to its anti-oxidant, antimutagenic and antitumor effects, galangin has been shown to possess anti-inflammatory, antimicrobial, and antiviral activities in a variety of *in vitro* and *in vivo* systems [3,4,5]. Furthermore, galangin has vasodilation [6], anti-ischemic and anti-oxidant properties, which might reduce the risk of coronary heart disease and improve endothelial cell function [7]. Li *et al.* [8] have shown that galangin has therapeutic potential as a neuroprotective agent for ischemic stroke. However, cerebral ischemia-induced neuronal injury is usually accompanied with significant changes in some neurotransmitters or metabolites, and the effect of galangin on the endogenous metabolites that were significantly changed in the brain tissue of rats with cerebral ischemia has not been explored yet.

Metabolomics, which refers to the comprehensive analysis of multiple endogenous metabolites, is becoming one of the most active research areas in biomedical systems [9]. Metabolomics attempts to capture the metabolic state of organisms by simultaneous and dynamic assessment of the small molecule metabolites in biological samples. Currently, several methods of standards including key biomarkers and metabolic pathways have been established in disease studies [10,11,12,13,14,15]. Because they act as essential neurotransmitters to control and regulate cerebral activity, amino acids, especially the excitatory amino acids (EAAs), which are responsible for several cerebral diseases [16], are always regarded as important biomarkers in metabolomics studies [17,18]. As one of the crucial analysis methods, amino acid metabolites have increasingly been focused on since they were first proposed by Noguchi *et al.* in 2003 [19]. To date, this technique has been extensively developed, particularly in the field of neurosciences and cerebral ischemia, for example, which is difficult to study with traditional methods. With the help of amino acid metabolites, the pathophysiologic processes of complex diseases such as cerebral ischemia, are gradually being studied [20,21]. Consequently, we choose the amino acid metabolomics method, which enables us to better understand pathological process and substance metabolic pathways in complex diseases, as our test method.

Although the method of tissue amino acid metabolomics has been used in cerebral ischemia diseases [22,23], this technology remains a novel method in ischemic stroke studies. In our previous studies [8,24,25], we found that galangin has therapeutic potential on ischemic stroke via regulation of serum amino acids in acute periods of cerebral ischemia. We further examined if galangin could also regulate tissue amino acids. Therefore, in the present study, by using the middle cerebral artery occlusion (MCAO) model, we report a novel attempt to explore the possible mechanism by which galangin improves acute ischemia stroke. In addition, we try to provide a better metabolites-transporter enzymes-protein network to help us better understand the stroke-related metabolic pathways.

## 2. Results

### 2.1. Method Validation

**Figure 1:**
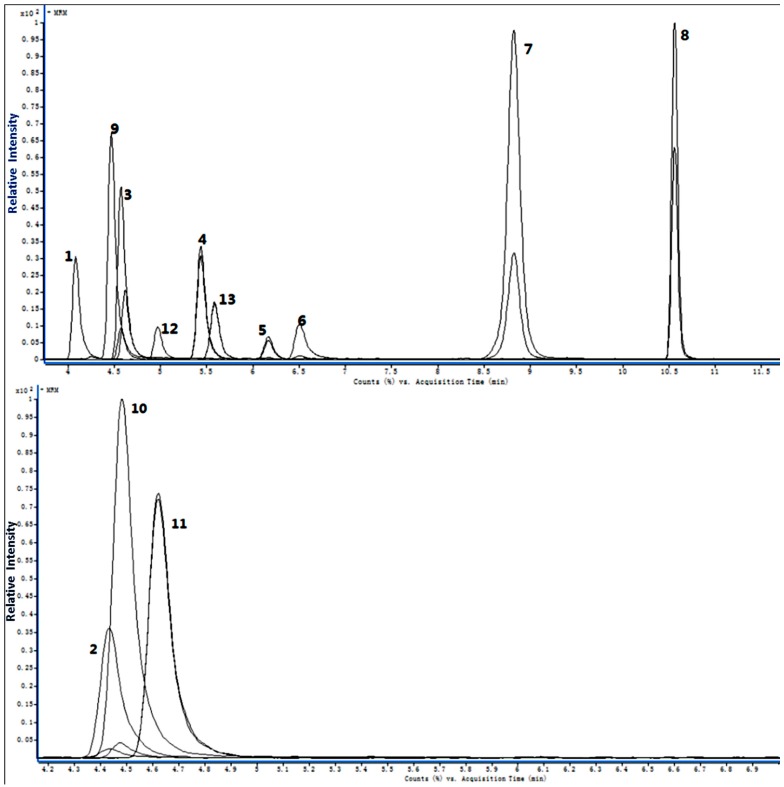
RRLC-MS/MS MRM chromatogram of the 12 AAs. 1, GABA; 2, Gly; 3, Glu; 4, Met; 5, Naa; 6, I.S.; 7, Phe; 8, Trp; 9, Ala; 10, Ser; 11, Asp; 12, Hcy; 13, Tyr.

The quantitative RRLC-QQQ analytical method was developed and validated to simultaneously determine the concentration of 12 endogenous AAs in rat brain tissue. The AA peaks were identified by comparisons of their retention times (Table 1) and based on the RRLC-MS/MS chromatograms of the 12 markers in Figure 1. The standard curves varied linearly and are shown in Table 2. The correlation coefficient (r^2^) for all analytes was above 0.9966, indicating good linearity. The limits of detection (LODs) were in the range of 0.02–0.64 ng·mL^−1^. The results of the calibration curves and LODs and limits of quantification (LOQs) values were summarized. The intra- and inter-day precision and accuracy of the method were determined from QC samples and are summarised in Table 3. The precision of the present method strictly conformed to the criteria for the analysis of biological samples where the relative standard deviation (RSD) determined at each level did not exceed 15%. The recoveries of the AAs during the sample preparation process were stable, and most of the recoveries ranged from 69.71% to 114.87%. The recovery for a biological sample must be at least 50% to be acceptable [26]. The results of all stability tests are presented in Table 4 and demonstrated a good stability of AAs over all steps of the determination at 4 °C. The samples were stable after three freeze/thaw cycles and frozen for 7 days at −80 °C. The RSD of reproducibility did not exceed 8.50%. The method, therefore, proved to be reliable and applicable to routine analysis.

### 2.2. Assessment of Neurological Defects

**Figure 2:**
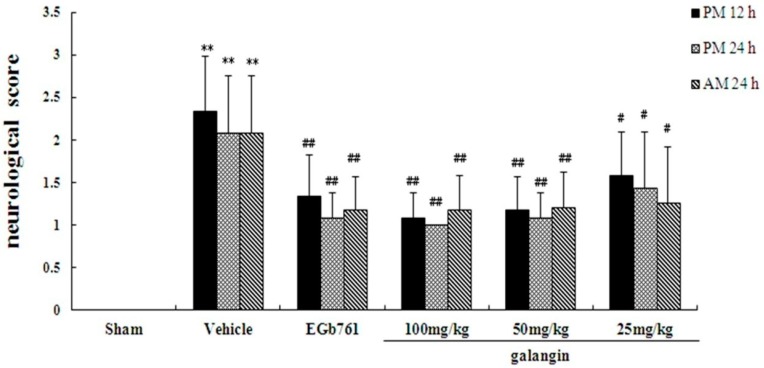
Effect of galangin on neurological deficits induced by MCAO and the neurological examination scores (behaviour test). In the neurological examination, a higher score indicates a worse pathological condition. The score of each experimental group is the average of ten subjects′ behavioural performances. The values are expressed as the means ± SD (*n* = 10), and the data were analysed using one-way ANOVA; ** *p* < 0.01 versus the sham groups, ^##^ *p* < 0.01, ^#^ *p* < 0.05 *vs.* the vehicle groups.

To reveal the effect of galangin on the neurological defects caused by the MCAO procedure, neurological defects were determined by a single researcher at 12 h and 24 h after MCAO. At PM 12 h, PM 24 h and AM 24 h, the mean neurological scores in the vehicle-treated groups were significantly (*p* < 0.01) higher than the mean scores in the sham groups, as shown in Figure 2, indicating a neurological defect after the MCAO operation. In the galangin-treated and positive control groups, the neurological deficits significantly improved (*p* < 0.05 or *p* < 0.01) compared with the vehicle-treated groups.

### 2.3. Effect of Galangin on the Level Changes of AAs in Rat Brain Tissue and Identification of Biomarkers

The levels of 12 endogenous amino acids (Glu, Trp, Phe, Tyr, Met, GABA, Asp, Ser, Ala, Naa, Gly and Asp) were determined at two different timepoints—12 h and 24 h—after the MCAO procedure. The results showed that the concentrations of all amino acids, except Naa, were increased in the tissue after the MCAO procedure; the Naa levels dropped significantly.

**Figure 3:**
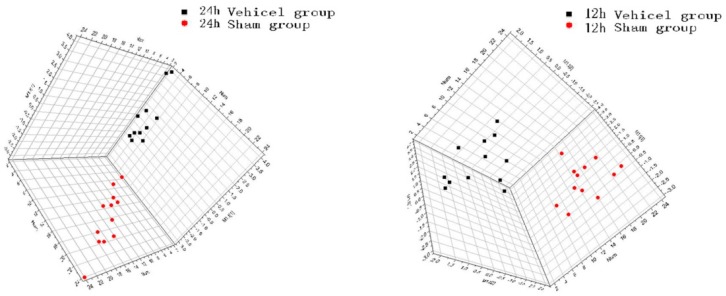
PLS-DA score plots derived from sham group and vehicle group of 12 h and 24 h MCAO by SIMCA-P12.0. In the score plots, the spots of the vehicle groups were clearly separated from the sham groups.

These 12 important endogenous amino acids that had remarkably altered concentrations between the vehicle and sham groups with PLS-DA (VIP > 1) were selected during this procedure. As the score plots show in Figure 3, there was a clear differentiation between the sham and vehicle groups at 12 h and 24 h after MCAO, which suggested that marked changes in the levels of amino acids occurred during the ischemic injury. Finally, we selected the AAs (Glu, Ala, Asp, GABA, Naa (12 h after MCAO), and Glu, GABA, Ser, Ala, Asp (24 h after MCAO)) as the result of PLS-DA.

**Figure 4:**
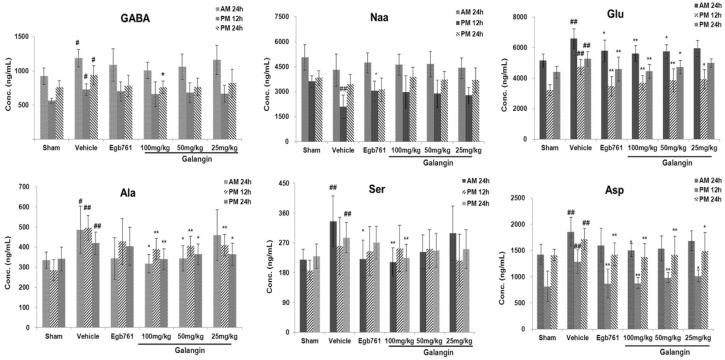
Effect of galangin on the levels of six amino acids (GABA, Naa, Glu, Ala, Ser and Asp). *n* = 10, means ± SD, ** *p* < 0.01, * *p* < 0.05 versus the sham group; ^##^ *p* < 0.01, ^#^ *p* < 0.05 *vs.* the vehicle control.

As shown in Figure 4, there were significant differences (*p* < 0.01 or *p* < 0.05) between the vehicle groups and sham groups in three AAs (Glu, Ala, Asp) at 12 h and 24 h after MCAO.

Galangin had a significant effect on the levels of three the AAs Glu, Ala and Asp in rats following MCAO. These three AAs were subsequently identified as the biomarkers that might play a role during the anti-cerebral ischemia mechanism of galangin. Further investigation of the bioinformatics analysis of metabolite-protein interaction networks was also performed in the following procedure.

### 2.4. Metabolite-Protein Interaction Networks

**Figure 5:**
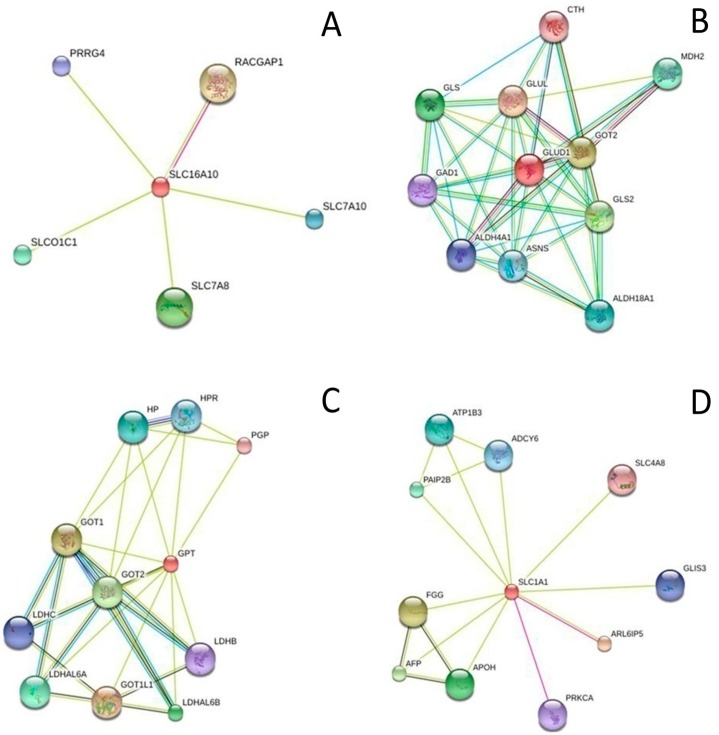
Functional interaction networks of SLC16A10 (**A**); GLUD1 (**B**); GPT (**C**) and SLC1A1 (**D**). The key enzymes (SLC16A10, GLUD1, GPT and SLC1A1) were collected and input into the STRING database to analyse the proteins that interacted with them. Only proteins with higher confidence were considered possible targets network for the metabolites. The protein was in turn docked into the protein targets. Each ligand-receptor pair would produce a MOE docking score, along with docking pose results, to represent the binding energy of this ligand-receptor complex.

**Figure 6:**
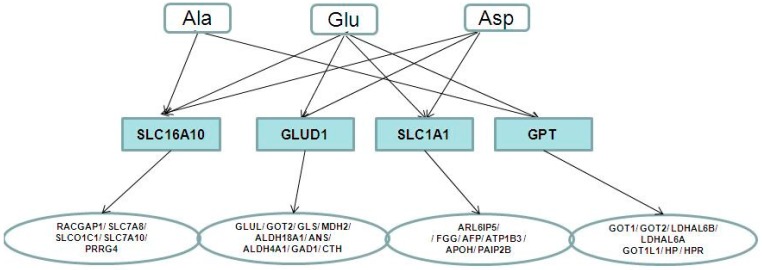
The three metabolites associated with stroke-related proteins. Metabolites, enzymes and proteins are represented with rounded rectangles, rectangles and ellipses, respectively.

The relationship between metabolites and proteins was analysed using metabolite-protein interaction networks to investigate the underlying molecular mechanism of the metabolites. Glu, Asp and Ala, which had already been reported to be closely related to stroke and significantly changed during our research, were used to find the metabolite-protein interaction networks. Four key enzymes (GLUD1, SLC16A10, SLC1A1 and GPT) related to the three AAs were collected using HMDB. With the STRING database, 28 cerebral ischemia-related proteins were linked to the three AAs through four key enzymes (Figure 5). As shown in Figure 6, the network of the links among the metabolites, enzymes and disease-related proteins were constructed.

**Figure 7:**
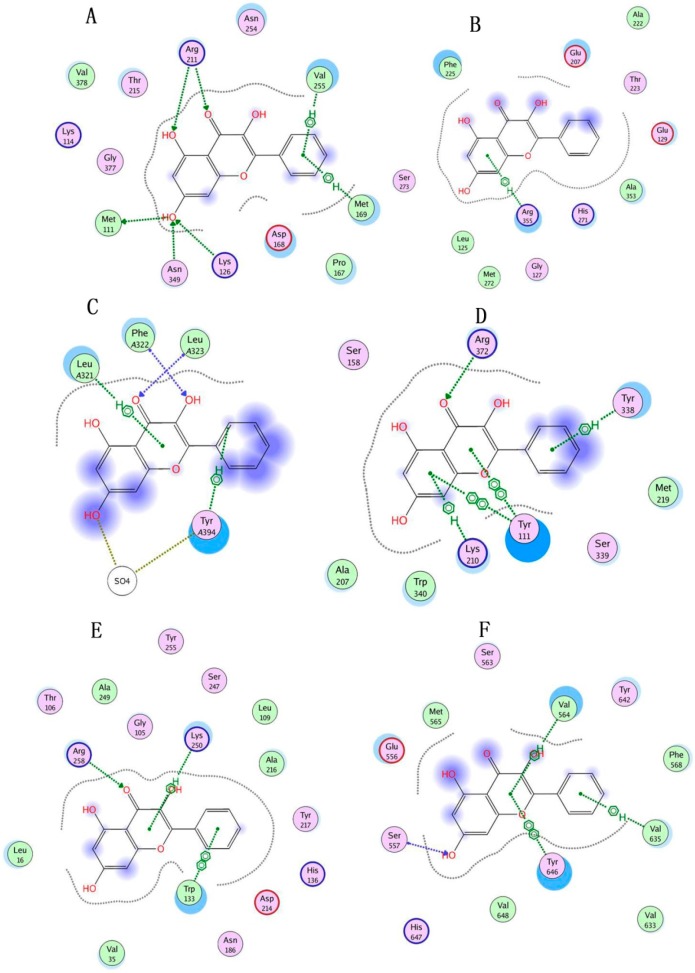
Molecular binding mode variation in the protein domains of galangin docking. (**A**) GLUD1; (**B**) GLUL; (**C**) GLS; (**D**) CTH; (**E**) GOT2; (**F**) HP.The different color rounds with different color ring outside stand for different character of the amino acid residue. The pink round with blue ring indicates that the amino acid residue is basic amino acid residue; the pink round with red ring stand for acidic one; the pink round with black ring infer that the amino acid residue is polar; the green round with black ring is greasy; and the blank round with black ring is metal complex. Arrow mark and green dotted line indicate hydrogen bonding and arene C-H bonding separately between galangin and protein domain residues. The blue shaded region indicates the solvent contacts made by galangin.

In the present study, molecular docking was performed to predict the probable targets of galangin against cerebral ischemia. The molecular docking of galangin into binding pockets revealed that the binding orientations were different among all of the proteins (Figure 7), and the docking results revealed a high affinity of galangin towards a few proteins (Table 5), especially GLUD1, GLUL, GLS, CTH, GOT2 and HP. Galangin was found to be interacting with the Arg 211, Val 255, Met 111, Asn 349, Lys 126 and Met 169 residues of GLUD1 with two arene-H bonds and two arene-arene bonds. GLUL was found to be interacting with galangin through Arg 355 residues with one arene-H bond. GLS showed interactions with galangin and Tyr A394, Leu A321, Phe A322 and Leu A323, forming two arene-H bonds. CTH showed an interaction with galangin at Lys 210, Tyr 111, Arg 372 and Tyr 338 residues forming two arene-arene bonds and two arene-H bonds. GOT2 was found to interact with galangin at Arg 258, Lys 250 and Trp 133 residues with one arene-H bond and one arene-arene bond. HP was found to be interacting with galangin at Ser 557, Tyr 646, Val 564 and Val 635 with two arene-H bonds and one arene-arene bond. The docking scores for the six proteins (−26.44535, −18.74692, −17.68718, −10.80142, −19.29626 and −19.11285 for GLUD1, GLUL, GLS, CTH, GOT2 and HP, respectively) were nearly that of their endogenous ligands and even lower. The scores indicate the strengths of the galangin-protein complexes, and lower values indicate greater stability.

## 3. Discussion

It was first proved that galangin had positive effects on the levels of AAs in brain tissue after ischemic stroke. Cerebral ischemiaresults from insufficient blood supply to a section of the brain, which in turn triggers various pathophysiological changes, including neurological defects and endogenous metabolic disorders. Herein, our results indicated that galangin at doses of 25, 50 and 100 mg/kg exhibited significant neuroprotective activity at 12 and 24 h after focal cerebral ischemiain a MCAO rat model both inthe PM and AM. And as a result, the improving effect of galangin at three dose levels on six selected AAs are no less than the positive control Egb761In the present study, a simple method based on RRLC-MS/MS technology was developed and validated to determine the concentration of endogenous AAs in rat brain tissue quantitatively and simultaneously. Some important AAs had remarkably altered concentrations between the vehicle and sham groups at 12 and 24 h after focal cerebral ischemia, and galangin had a significant effect on the AAs levels in rat brain tissues. With PLS-DA, multivariate statistics could differentiate MCAO and sham rats. Five AAs (Glu, Ala, Asp, GABA, Naa) at 12 h after MCAO and five AAs (Glu, GABA, Ser, Ala, Asp) at 24 h after MCAO had VIP > 1. Glu, Ala and Asp had significantly altered tissue concentrations between the galangin-treated (50 and 100 mg·kg^−1^) groups and vehicle groups and were selected as the biomarkers of galangin in cerebral ischemia. These AAs have already been reported to participate in the process of cerebral ischemiaand were used to screen the pathway-related enzymes that were reported to be relevant to stroke with HMDB [24]. Finally, four key enzymes (GLUD1, SLC16A10, SLC1A1 and GPT) related to the three AAs (Glu, Ala and Asp) were collected. In our results, the four enzymes might participate in the process of cerebral ischemiaand galangin anti-cerebral ischemiathrough the metabolism of amino acids in brain tissue. With the STRING database, 28 proteins were linked to the three AAs (Glu, Ala and Asp) through the four enzymes, and the links among the AAs, enzymes and proteins were explored. With the docking design to galangin, the six proteins (GLUD1, GLUL, GLS, CTH, GOT2 and HP) with high affinity might become its targets for cerebral ischemia. In our research, the high affinity of GLUL to galangin as its target for cerebral ischemiawas found in serum [27] and brain tissue samples, and it was reported that the oxidative damage-induced loss of GLUL activity might be a key event in stroke-induced brain injury [24].

We found that galangin plays a protective effect on ischemic stroke through the analysis of metabolites in rat brain tissue. The anti-cerebral ischemiaeffect of galangin might be correlated with the three AAs (Glu, Ala and Asp) that participated in the regulation of the signalling pathway of four stroke-related enzymes.

## 4. Materials and Methods

### 4.1. Chemicals and Reagents

l-Glycine (Gly), alanine (Ala), γ-aminobutyric acid (GABA), l-aspartic acid (Asp), l-glutamic acid (Glu), L-methionine (Met), l-phenylalanine (Phe), tyrosine (Tyr), d-serine (d-Ser), homocysteine (Hcy), *N*-acetylaspartic acid (Naa), and tryptophan (Trp) were purchased from Sigma Aldrich Co., Ltd. (Shanghai, China). Formic acid (purity 99%) was obtained from Roe Scientific Inc. (Newark, DE, USA). All solutions were prepared using LC-MS Ultra High Purity water and LC-MS-grade acetonitrile (Tedia Company Inc., Fairfield, OH, USA). The I.S. (acrylamide-*d*_3_) was purchased from Toronto Research Chemicals Inc. (Toronto, ON, Canada).

Galangin with 98.0% purity was purchased from Nanjing Zelang Medical Technology Co., Ltd (Nanjing, China). The stock solution was prepared by dissolving the galangin power in sterile saline (containing 5% polysorbate 80). EGb761 (Folium Ginkgo Extract), used as a positive control to explore the efficacy of galangin, was purchased from Dr. Willmar Schwabe (Karlsruhe, Germany).

### 4.2. Animals

All animal experiments were performed on male Sprague-Dawley rats (Beijing Vital River Company, Beijing, China) weighing 250–270 g, housed individually at 22 ± 2 °C with a relative humidity of 50% ± 10%, a 12-h day-night cycle and free access to chow and water. The rats were allowed to adapt to the housing conditions for 2 days before the experiments were performed. All animal experiments were performed in accordance with institutional guidelines and ethics. The Institute of Chinese Materia Medica, China Academy of Chinese Medical Sciences approved all animal experiments performed in this study.

### 4.3. Animal Models and Experimental Protocol

According to the protocol of Longa [28] with slight improvements, the MCAO procedure was carried out at two different timepoints (12 h and 24 h). Rats were performed anesthesia with chloral hydrate (10%, 400 mg·kg^−1^) and placed in a supine position on an operation table. The rectal temperature was recorded and maintained at 37 ± 0.5 °C throughout the surgical procedure. A midline neck incision was made. The left common carotid artery, the left external carotid artery and the left inner carotid artery were exposed. A fishing thread (diameter of 0.26 mm) lightly dipped in paraffin was inserted into the left common carotid artery and occluded the middle cerebral artery. The animals in the sham group underwent the same procedures as described above with the exception of the insertion of the nylon filament into the inner carotid.

Considering the time and dose-response study, we randomly divided the rats into the following six groups (*n* = 10/group): sham group, vehicle control group, positive control EGb761 group (4 mg·kg^−1^), and galangin-treated groups (low, middle and high dosage: 25, 50 and 100 mg·kg^−1^, respectively). The rats in the sham group (S) underwent the same procedures as described above except the insertion of the nylon filament into the inner carotid. Galangin and the positive control EGb761 were administered intragastrically (i.g.) 15 min prior to MCAO for 12 h (PM 12 h), 15 min prior to MCAO for 24 h (PM 24 h) and 6 h after MCAO for 24 h (AM 24 h). The sham and vehicle-treated rats were given physiological saline intragastrically under the same protocol. The neurological defects were determined 12 h and 24 h after the MCAO procedure.

### 4.4. Assessment of Neurological Defects

Neurological defects were evaluated 12 h and 24 h after the MCAO procedure using a score scale modified from Huang *et al.* [29], and then the animals were sacrificed. The neurological scores were defined as follows: 0, no neurological deficit; 1, failure to extend the right forelimb; 2, circling to the contralateral side; 3, falling to the contralateral side at rest; and 4, no spontaneous motor activity.

### 4.5. Brain Tissue Homogenate Sampling and Preparation

The brain samples were weighed and homogenised in 5 mL of initial mobile phase (90% water with 0.1% formic acid and 10% acetonitrile) prepared in cold (0 °C) using a homogeniser. The brain homogenate was collected in a 10 mL centrifuge tube and was then centrifuged at 3000 rpm for 10 min at 4 °C. The supernatant was maintained in the dark at −80 °C until analysis, and 25 μL of the supernatant was transferred into a 1.5 mL Eppendorf tube and spiked with 10 μL of I.S. stock solution (final concentration 1000 ng·mL^−1^), then the initial mobile phase was added, and the final volume was 500 μL. After vortex mixing for 1 min, the mixture was centrifuged at 12,000 rpm for 10 min at 4 °C and then filtered, and the supernatant was injected into the HPLC-MS analysis.

### 4.6. Instrumentation (Chromatographic and MS Spectrum Conditions)

Rapid resolution liquid chromatography (RRLC) analysis was performed on an Agilent 1200 rapid resolution liquid chromatography instrument (Agilent, Santa Clara, CA, USA) equipped with an autosampler, a binary pump, an online vacuum degasser and a thermostated column compartment. The analytical column was a Diamonsil C18(2) column (5 µm, 250 mm × 4.6 mm) maintained at 30 °C column temperature. The mobile phase for elution was a gradient established between solvent A (water containing 0.1% formic acid) and solvent B (acetonitrile) at 0.5 mL·min^−1^. Baseline separation was achieved using a gradient starting from 90% A/10% B followed by a linear increase of B, reaching 50% B at 10 min. The eluent was returned to the initial conditions over 20 min, after which the eluent was maintained until the end of the run to allow equilibration.

An Agilent G6410 triple quadrupole mass spectrometer equipped with an electrospray ion (ESI) source (Agilent, Lexington, MA, USA) was operated in positive ion mode using MRM scanning. The following optimal conditions were used: electrospray capillary voltage, 4000 V; nebuliser pressure, 45.0 psi; drying gas, nitrogen; flow rate, 11 L/min; and temperature, 350 °C. High-purity nitrogen was used as the collision gas. Standard solutions were infused into the MS for optimisation to establish the appropriate MRM conditions. The acquisition parameters for each analyte are listed in Table 1. The fragmentor voltage (FV) and collision energy (CE) varied among the different markers. Agilent Mass Hunter workstation software version B.01.04 was used for the data acquisition and processing.

### 4.7. Identification of the Endogenous AAs

In our research, all of the parameters including linearity, inter- and intra- assay precision, recoveries, sensitivity, and stability limit of detection (LOD) and limit of quantification (LOQ) were evaluated to establish and validate a rapid, sensitive and accurate methodology of simultaneous quantification of 12 AAs in rat brain tissue by the RRLC/QQQ. The sensitivity of the method was determined by quantifying the LOD and LOQ for each compound. The LOD and LOQ were evaluated by considering the analyte concentrations that yield a signal- to- noise ratio (S/N) of 3 and 10, respectively. Calibration was performed with a least-squares linear regression of the peak area ratios of the AAs to the I.S. versus the respective standard concentration. The intra-day and inter-day precision were studied by determination at three different concentrations of QC samples. The intra-day precision was assessed by repeatedly analysing the AAs in the QC samples (*n* = 6) in a day. The inter-day precision was evaluated by repeated analysis of AA in the QC samples over three consecutive days. The recovery was calculated based on the difference between the total concentration determined in the spiked samples and the concentration in the non-spiked samples. The stability of AAs in rat brain tissue was assessed by analysing the replicates (*n* = 6) of samples during the same storage and processing procedures. The collected tissue samples were analysed with commercially purchased standards.

### 4.8. Data Analysis

All data were acquired and processed using Agilent Mass Hunter workstation software version B.01.04 (Agilent, Santa Clara, CA, USA). The quantitative data are presented as the means ± SD. The data of vehicle groups were compared with the data of sham groups, and the other groups were compared with the vehicle groups, respectively. The Kolmogorov-Smirnov test was used to determine the normality of the distribution of continuous variables. The significance of variables was determined using one-way ANOVA. PLS-DA was used to explore the difference between the sham and vehicle group. The variable importance in projection (VIP > 1), which was selected for the cutoff value for searching the most important variables [30], was used to extract novel potential biomarker ions in the PLS-DA model. PLS-DA analyses were applied with SIMCA-P 12.0 software (Umertrics, Umea, Sweden).

### 4.9. Network Analysis

Significantly different AAs between the galangin-treated groups and vehicle-treated groups were further analysed. The AAs were input into the HMDB, the corresponding HMDB IDs were retrieved, and then key enzymes related to each AA were collected. The common key enzymes that were related to two or more AAs were discovered and input into the Search Tool for the Retrieval of Interacting Genes/Proteins (STRING database) [31] to analyse the proteins that interacted with them. Only proteins with high confidence were considered possible target networks for the metabolites [32].

### 4.10. Molecule Docking

Molecular docking was performed using the molecular operating environment (MOE) docking software (MOE 2010). The 2D structure of galangin was downloaded from PubChem (http://pubchem.ncbi.nlm.nih.gov), and the energy of the 3D geometry was minimised in the MOE working environment. The structures of 8 proteins with endogenous ligands were obtained from the Protein Data Bank (PDB) (Table 6). Hydrogens and partial charges were added to the structures of eight crystal complexes with the protonated 3D application. The docking site was defined by the ligand in every crystal complex. We used Triangle Matcher as the placement method. The docked conformations were ranked by the London dG scoring function, which estimates the free energy of ligand binding from a given conformation. The docking method was validated by the value of the root-mean-squared-deviation (RMSD) between the heavy atoms of the predicted pose and the value of the ligand of the co-crystal structure. The conformations were refined and rescored with the force field method to obtain more reasonable conformations. At the end of docking, the binding modes of galangin to 8 proteins were observed to identify the important residues.

## 5. Conclusions

Our results provide new evidence of the potential therapeutic effects of galangin on the changes of AAs in the treatment of ischemic stroke in a dose-dependent manner at PM and AM time points. This paper was designed to study the metabolomics characteristics of cerebral ischemia and the therapeutic effect of galangin by constructing a link of the downstream metabolites, enzymes and proteins. We also demonstrated that metabolic profiling might be a useful tool to discover biomarkers and envision a holistic view of metabolism for cerebral diseases.

## Figures and Tables

**Table 1:** Ion transitions for AAs and instrumental parameters for MS-MS detection in MRM mode.

AAs + I.S.	*m*/*z*	Ion Transition	Dwell Time (s)	FV (V)	CE (eV)	Mean RT
Naa	176.1	176.1 > 134.3	20	80	0	6.176
		176.1 > 88.1	20	80	10	6.176
Ala	90.1	90.1 > 44.2	10	45	10	4.466
GABA	104.1	104.1 > 87.2	20	80	10	4.077
Phe	166.3	166.3 > 120.3	10	90	10	8.833
		166.3 > 103.2	10	90	20	8.833
Tyr	182.3	182.3 > 165	10	110	4	5.579
		182.3 > 136.3	10	110	10	5.579
Gly	76.2	76.2 > 48.2	10	50	3	4.432
		76.2 > 30.2	10	50	4	4.432
Met	150.2	150.2 > 133	10	55	4	5.432
		150.2 > 104.2	10	55	5	5.432
Trp	205.2	205.2 > 188.3	10	90	7	10.535
		205.2 > 146.2	10	90	17	10.535
Asp	134.2	134.2 > 88.2	10	60	5	4.620
		134.2 > 74.2	10	60	12	4.620
Ser	106.1	106.1 > 60.2	10	70	10	4.478
		106.1 > 42.2	10	70	10	4.478
Glu	148.2	148.2 > 102	10	90	4	4.570
		148.2 > 84.2	10	90	10	4.570
Hcy	136.1	136.1 > 90.2	20	80	10	4.966
		136.1 > 56.2	20	80	20	4.966
acrylamide-*d*_3_	75.1	75.1 > 58.1	10	40	8	6.505
		75.1 > 30.2	10	40	8	6.505

**Table 2:** Quantitative parameters for analysis of AAs in brain tissue.

Chemicals	Equations	Correlation Coefficient (r^2^)	Linear Range ^a^ (ng·mL^−1^)	LOD ^b^ (ng·mL^−1^)	LOQ ^c^ (ng·mL^−1^)
Ala	y = 3.0611x + 0.0165	0.9999	10–5000	0.53	1.79
GABA	y = 1.1833x − 0.0018	0.9991	10–5000	0.31	1.05
Ser	y = 1. 0241x + 0.0230	0.9997	10–5000	0.49	1.67
Asp	y = 0.6314x + 0.0040	0.9997	10–5000	0.49	1.67
Gly	y = 0.5793x + 0.0018	0.9990	10–5000	0.51	1.73
Hcy	y = 0.3371x + 07.73583 × 10^−4^	0.9966	1–500	0.10	0.35
Glu	y = 1.5217x + 0.0102	0.9999	20–10000	0.47	1.59
Met	y = 1.7855x + 0.0055	0.9993	1–500	0.05	0.18
Phe	y = 7.4569x + 0.0922	0.9981	1–500	0.02	0.07
Naa	y = 0.0789x − 2.50132 × 10^−4^	0.9994	20–10000	0.64	2.17
Tyr	y = 0.8392x + 0.0126	0.9981	1–500	0.05	0.18
Trp	y = 4.6319x + 0.0199	0.9996	1–500	0.13	0.43

^a^ The calibration curves were constructed using relative responses versus the relative concentration of each analyte. Each calibration curve was derived from seven data points (*n*= 6), except Hcy (*n* =7). ^b^ LOD refers to the limit of detection in serum in ng·mL^−1^. ^c^ LOQ refers to the limit of quantification in serum in ng·mL^−1^.

**Table 3:** Determination of neurotransmitter concentrations by HPLC-MS/MS: validation results for precision and recovery.

Chemicals	Intra-Day Precision RSD (%, *n* = 6)			Inter-Day Precision RSD (%, *n* = 6)			Recovery (%, *n* = 6)
	Low	Mid	High	Low	Mid	High	
GABA	0.54	1.69	8.03	1.69	3.25	8.18	98.80
Gly	3.64	1.81	6.22	4.31	3.13	6.13	97.46
Ala	2.95	1.72	8.10	3.62	2.90	6.36	96.21
Ser	3.43	1.64	8.39	4.55	3.29	8.20	108.58
Glu	1.60	2.47	8.35	2.33	3.51	7.05	100.38
Asp	6.63	2.39	8.65	5.32	4.07	6.62	85.11
Hcy	3.78	3.99	7.56	4.66	4.91	9.42	114.87
Met	7.86	3.02	7.02	7.89	4.03	8.91	78.78
Tyr	10.97	4.11	7.52	9.73	6.60	9.58	69.71
Naa	3.07	1.69	9.16	2.87	3.41	9.25	107.96
Phe	5.70	3.36	8.43	6.16	3.24	6.77	72.10
Trp	8.72	6.57	7.99	6.41	7.20	9.83	75.72

**Table 4:** Determination of amino acids by HPLC-MS/MS: validation results on stability and repeatability.

Chemicals	Stability RSD (%, *n* = 6)				Repeatability RSD (%, *n* = 6)
	−80 °C Freeze	Freeze-Thaw	Room Temperature	4 °C	
GABA	6.44	9.37	11.22	9.54	5.72
Gly	11.53	7.16	15.32	5.08	3.75
Ala	12.20	6.45	15.42	4.33	3.65
Ser	12.72	6.73	15.98	6.13	3.48
Glu	6.19	6.89	13.82	3.63	3.62
Asp	9.49	6.66	13.60	3.43	2.88
Hcy	16.90	8.07	6.51	8.75	8.35
Met	14.59	5.54	13.51	4.81	3.00
Tyr	11.85	9.48	15.07	7.28	6.61
Naa	12.52	6.60	6.70	4.61	3.22
Phe	9.47	4.87	13.70	3.90	3.26
Trp	8.95	5.63	7.84	5.02	2.90

**Table 5:** Molecular docking of galangin into the binding pockets of related proteins.

Proteins	MOE Docking of Endogenous Ligands		MOE Docking Scores of Galangin	Interacting Residues
	RMSD	Scores		
GLUD1	1.256284	−22.78383	−26.44535	Arg 211, Val 255, Met 111, Asn 349, Lys 126, Met 169
GLUL	0.9439976	−35.19292	−18.74692	Arg 355
GLS	1.326625	−21.21566	−17.68718	Tyr A394, Leu A321, Phe A322, Leu A323
GAD1	3.128657	−16.44727		
CTH	1.960125	−16.61392	−10.80142	Lys 210, Tyr 111, Arg 372, Tyr 338
ATP1B3	3.068527	−13.54975		
GOT2	1.691135	−21.93334	−19.29626	Arg 258, Lys 250, Trp 133
HP	1.764202	−15.15395	−19.11285	Ser 557, Tyr 646, Val 564, Val 635

**Table 6:** The abbreviations, full names and PDB numbers of 8 proteins.

Proteins	Full Name	Pdb Number
GLUD1	Glutamate dehydrogenase 1, mitochondrial	3ETD
GLUL	Glutamine synthetase	1FPY
GLS	Glutaminase	3VOZ
GAD1	Glutamate decarboxylase 1	4HKP
CTH	cystathionase	2FQ6
ATP1B3	ATPase	3N23
GOT2	Aspartate aminotransferase	1IVR
HP	haptoglobin	3QUG
